# Post hysteroscopic progesterone hormone therapy in the treatment of endometrial polyps

**DOI:** 10.12669/pjms.345.15330

**Published:** 2018

**Authors:** Fangfang Li, Shuangyan Wei, Shuye Yang, Zhiqiang Liu, Fangfang Nan

**Affiliations:** 1 Fangfang Li, Department of Obstetrics and Gynecology, Binzhou Medical University Hospital, Binzhou, 256603, China; 2 Shuangyan Wei, Department of Obstetrics and Gynecology, Binzhou Medical University Hospital, Binzhou, 256603, China; 3 Shuye Yang, Department of Orthopaedics, Binzhou Medical University Hospital, Binzhou, 256603, China; 4 Zhiqiang Liu, Department of Obstetrics and Gynecology, Binzhou Medical University Hospital, Binzhou, 256603, China; 5 Fangfang Nan, Department of Obstetrics and Gynecology, Binzhou Medical University Hospital, Binzhou, 256603, China

**Keywords:** Endometrial polyp, Hysteroscope, Progesterone hormone

## Abstract

**Objective:**

To find out the clinical effects of post hysteroscopic progesterone hormone therapy in the treatment of endometrial polyps in terms of clinical outcome and the expression of endometrial Vascular Endothelial Growth Factor (VEGF).

**Methods:**

Ninety-eight patients who were confirmed as endometrial polyp in the hospital from April 2014 and December 2016 were selected and divided into treatment group and a control group using random number table, 49 in each group. Patients in both groups were given hysteroscopic operation. Patients in the treatment group were treated by progesterone hormone drugs after hysteroscopic operation, while patients in the control group were not given progesterone hormone. The changes of menstrual blood volume, menstrual cycle and expression of VEGF were compared between the two groups after treatment, and the recurrence condition, thickness of endometrium and hemoglobin were followed up one year after treatment.

**Results:**

The pictorial blood loss assessment chart (PBAC) scores of patients in the two groups had no significant difference before treatment (P>0.05); but the score of the treatment group was much lower than that of the control group. The improvement rate of menstrual cycle of the treatment group was much higher than that of the control group, and the difference had statistical significance (P<0.05). Compared to before treatment, the serum VEGF level of the patients in both groups had a remarkable decline in the 1^st^, 3^rd^ and 6^th^ month after treatment, and the difference had statistical significance (P<0.05). The difference of the serum VEGF level between the two groups in the 1^st^ and 3^rd^ month after treatment had no statistical significance (P>0.05). The serum VEGF level of the treatment group was notably lower than that of the control group six months after treatment, and the difference had statistical significance (P<0.05). The follow-up results demonstrated that the treatment group had smaller thickness of endometrium and higher level of hemoglobin compared to the control group, and the recurrence rate of the treatment group was lower than that of the control group (P<0.05).

**Conclusion:**

Post hysteroscopic progesterone hormone therapy has favorable clinical effect in treating endometrial polyps as it can effectively prevent the recurrence of endometrial polyps, relieve the level of hemoglobin and reduce endometrial thickness.

## INTRODUCTION

Endometrial polyp, one of the common gynecological diseases, has an incidence of 5.7% in China.^1^ It was reported that endometrial polyp was induced by local hyperplasia of endometrium, and its common clinical manifestations included single or multiple smooth mass with pedicles in different lengths standing out from uterine cavity which can induce excessive menstrual blood volume or irregular metrorrhagia.^2^ Endometrial polyp mostly occurs in middle-aged women. Delayed treatment or improper treatment method may cause adverse impacts on function of uterus and even induce infertility.^3^

Transcervical Resection of Polyp (TCRP) under hysteroscopy, a minimally invasive effective method for treating endometrial polyp, is considered as a gold standard for treatment of endometrial polyp. TCRP^4^ has little damages to endometrium and can effectively relieve menstrual disorder.^5^ But TCRP alone cannot thoroughly resect endometrial polyp and is unable to effectively inhibit the supply of blood to endometrial polyp, leading to higher recurrence rate.^6^ Previous studies have suggested that short-acting or slowly released progesterone hormone in combination with hysteroscopic resection was significantly effective in preventing postoperative recurrence of endometrial polyp.^7^-^9^

This study mainly investigated the clinical effect of post hysteroscopic progesterone hormone therapy in the treatment of endometrial polyps, deeply studied whether the combined therapy could effectively control the recurrence of endometrial polyp, identify the changes of the serum level of Vascular Endothelial Growth Factor (VEGF) before and after treatment, and explored the possible mechanism of the formation of endometrial polyp, which provides a reliable and effective evidence for the clinical treatment of endometrial polyps.

## METHODS

Ninety-eight patients who were admitted to the hospital from April 2014 to December 2016 were selected. Patients were included if they were confirmed as endometrial polyp by hysteroscopic or color dopplar ultrasound examination, was not allergic to progesterone hormone drugs, and did not take hormone drugs in recent half year. Those who had dysfunction of the heart, liver, lung and kidney, malignant tumor, had been menopausal, and had contraindications to hysteroscopic examination and treatment were excluded. They were divided into a treatment group (N=49) and a control group (N=49) using random number method. In the treatment group, the patients aged from 23 to 64 years (average 39.7±6.4 years) and had a disease course of 18 d to 38 months (average 13.7±3.6 months); there were 19 cases of menstrual disorder, 14 cases of abnormal vaginal bleeding, seven cases of hemorrhagic leucorrhea and nine cases of stomachache. In the control group, the subjects aged from 22 to 63 years (average 37.3±7.2 years) and had a disease course of 20 d to 41 months (average 15.3±3.2 months); there were 21 cases of menstrual disorder, 12 cases of abnormal vaginal bleeding, seven cases of hemorrhagic leucorrhea and nine cases of stomachache. The difference of general data between the two groups had no remarkable difference (P>0.05). written informed consent was taken. The study has been approved by the medical ethical committee of the hospital.

### Therapeutic method

TCRP of the two groups was performed by the same doctor. The uterine neck was softened by misoprostol before surgery. The surgery was performed under compound intravenous anesthesia. Firstly the number, position and size of lesions were probed under hysteroscope. Then lesions were thoroughly resected using the ring electrode of the hysteroscope at a power of 75 ~ 90 W. The resection parts included the basilar parts of lesions and their surrounding tissues. The patients orally took anti-infective drugs after surgery. Patients in the control group were not given any drug treatment to prevent recurrence after surgery, while patients in the treatment group orally took dydrogesterone tablets (brand name: Dafutong; Netherlands Abbott Healthcare Products B. V.; registration number: H20110211) one week after operation, in a dose of 10 mg, twice a day, for 10 days. After the treatment, each patient took it in the 5^th^ day of menstrual cycle, continuously for 21 days; the course was four months.

### Outcome measure

The improvement of menstrual blood volume and menstrual cycle of the two groups was observed after treatment (menstrual blood volume was evaluated using pictorial blood loss assessment chart (PBAC)). They were followed up for six months after operation. The recurrence conditions in the two groups were also observed; patients whose ultrasonic examination results suggested abnormal echo in the uterine cavity and results of hysteroscopy and pathological examination suggested endometrial polyp were considered having recurrence. Transvaginal ultrasonic examination content included endometrial thickness (measured in the 24^th^ and 29^th^ day in a cycle) and hemoglobin; the examination was performed by the same doctor to avoid subjective error. 5 mL of fasting peripheral venous blood was extracted 24 hour before operation and in the 1^st^, 3^rd^ and 6^th^ month after operation and centrifuged at 3000 r/min for 10 minutes to separate serum. The obtained serum was preserved separately at -20°C. Double antibody enzyme-linked immunosorbent assays (ELISA) was used to detect the level of serum VEGF. Kit (SYNTRON Company, USA) was used. The operation strictly followed the instructions.

### Statistical method

Data were analyzed using SPSS ver. 20.0. Numerical data were expressed as mean ± standard deviation and processed by t test. Categorical data were expressed as percentage (%) and processed by Chi-square test. Difference had statistical significance if P<0.05.

## RESULTS

### The PBAC score and improvement rate of menstrual cycle of the two groups

The difference of the PBAC score between the two groups was not statistically significant before treatment (P>0.05); but the PBAC score of the treatment group was significantly lower than that of the control group after treatment. The menstrual cycle of the treatment group was remarkably higher than that of the control group, and the difference had statistical significance (P<0.05; Table-I).

**Table-I:** PBAC score and the improvement rate of length of menstrual cycle

Group	PBAC score before treatment (point)	PBAC score after treatment (point)	Improvement rate of menstrual cycle (%)
Treatment group	220.5±45.2	50.2±10.2	44(89.8)
Control group	218.6±46.0	72.5±12.0	32(65.3)
t/X^2^	3.274	11.197	12.363
P	>0.05	<0.05	<0.05

### The level of serum VEGF of the two groups before surgery and in postoperative follow up

Compared to before treatment, the level of serum VEGF of both groups significantly declined in the 1^st^, 3^rd^ and 6^th^ month after treatment, and the difference had statistical significance (P<0.05). The difference of the level of serum VEGF between the two groups in the 1^st^ and 3^rd^ month after operation was not statistically significant (P<0.05). The level of serum VEGF of the treatment group was apparently lower than that of the control group in the 6^th^ month after operation, and the difference was statistically significant (P<0.05; Table-II).

**Table-II:** Level of serum VEGF between the two groups before and after intervention

Group	VEGF level (ng/L)			

	Before operation	1^st^ month after operation	3^rd^ month after operation	6^th^ month after operation
Treatment group	155.3±45.2	30.8±6.6*	39.6±8.3*	45.3±11.1*^#^
Control group	157.7±42.3	38.3±5.8*	55.4±10.2*	81.2±15.7*

* Note: indicated P<0.05 compared to before operation;

# indicated P<0.05 compared to the control group.

### The changes of endometrial thickness and hemoglobin of the two groups before and after surgery

The follow-up results of the 6^th^ month suggested that the changes of endometrial thickness and hemoglobin of the treatment group was significantly superior to those of the control group, and remarkable differences were observed (P<0.05; Table-III).

**Table-III:** Endometrial thickness and hemoglobin between the two groups before operation and six months after operation.

Group	Endometrial thickness (mm)		Hemoglobin (g/L)	
	
	Before operation	6^th^ month after operation	Before operation	6^th^ month after operation
Treatment group	10.69±2.51	5.79±1.42	88.49±10.25	108.32±15.37
Control group	10.84±2.53	7.64±1.57	88.51±10.26	96.84±6.56
t	1.025	3.276	1.023	4.134
P	>0.05	<0.05	>0.05	<0.05

### The recurrence condition of the two groups during follow up after operation

Only one case in the treatment group recurred (2.04%); six cases in the control group recurred (12.24%). The recurrence rate of the treatment group was lower than that of the control group, indicating a remarkable difference (X^2^=28.174, P<0.05).

## DISCUSSION

Endometrial polyp, a common benign gynecological disease can occur in females from any age group. Many factors can lead to endometrial polyp, such as inflammatory reaction, change of endocrine function and estrogen secretion.^10^ Women during reproductive age group are at increased of endometrial polyp with varied clinical manifestations like menorrhagia and subfertility.^11^

Currently surgical resection is the main method for treating endometrial polyp. With the rapid development of endoscopic techniques, hysteroscopic electrocision has gradually been an important approach in the treatment of endometrial polyp. The number, structure and surface blood supply of endometrial polyps can be clearly seen under hysteroscope;^12^-^14^ moreover polyps can be thoroughly removed by electrotome, and the depth of resection can be controlled to reduce damages to body and benefit postoperative recovery. However evidence suggests chances of recurrence after hysteroscopic resection. A clinical study demonstrated that the long-term recurrence rate of TCRP under hysteroscope was 25%, which might be correlated to the levels of hormones.^15^,^16^ Thus to effectively reduce and prevent postoperative recurrence after TCRP, postoperative oral administration of drugs can be given to patients to prevent polyps regeneration.

It was pointed out that the prevention measures for the recurrence of endometrial polyp were diversified, including progesterone hormone preparations, short-acting contraceptives, gestrinone, gonadotrophin releasing hormone, traditional Chinese medicines and intrauterine devices containing sustained-release drugs.^17^ A study of Pukkala et al. found that progesterone hormone at a large dose could efficiently act on endometrium to suppress endometrial hyperplasia, relieve the symptom of abnormal vaginal bleeding after vasoconstriction and prevent recurrence of endometrial polyp.^18^ A study explored preventive treatment on patients with endometrial polyp after surgery;^19^ after drug treatment, the menstrual cycle became regular, and menstrual blood volume significantly reduced, suggesting that postoperative administration of drugs could effectively regulate menstrual cycle and menstrual blood volume. The results of this study indicated that the treatment group had superior improvement of the menstrual cycle and menstrual blood volume than the control group. Moreover, another study found that patients with endometrial polyp had anemia; patients who took progesterone hormone drugs had significantly increased level of hemoglobin and relieved anemia; the endometrial thickness reduced after drug administration, and the increase of thickness became slower. The follow-up results suggested that the recurrence rate of the patients who received hysteroscopic progesterone hormone therapy was lower than that of patients who underwent surgery merely. The above findings were consistent with the results of this study.

VEGF with a high specificity plays a vital role in the occurrence and development of diseases. A study found that VEGF could specifically promote neovascularization and vascular endothelial cell division, accelerate the growth of blood vessels in endometrial polyps, and increase blood supply.^20^ This study demonstrated that the serum VEGF level of the patients in both groups significantly decreased in the 1st, 3rd and 6th month after treatment compared to before surgery. The serum VEGF level of the patients who were treated by dydrogesterone tablets in combination with TCRP was remarkably lower than that of the patients who was treated by TCRP only in six-month follow up, indicating the serum of the patients with endometrial polyps had high expression of VEGF and both therapies could effectively reduce the expression of serum VEGF. However, dydrogesterone tablets in combination with TCRP were more effective. It also indicated that the decline of serum VEGF level can provide an evidence for the reasonability of the treatment scheme.

## CONCLUSION

Post hysteroscopic progesterone hormone therapy is an effective strategy in the clinical treatment of endometrial polyp. It can improve the postoperative level of hemoglobin, reduce thickness of endometrium, and effectively prevent postoperative recurrence.

### Authors’ Contribution

**FFL & SYW:** Study design, data collection and analysis.

**FFL, SYY, ZQL & FFN:** Manuscript preparation, drafting and revising.

**FFL & SYW:** Review and final approval of manuscript.
